# Protective mechanical ventilation in suspected influenza infection

**DOI:** 10.1590/0037-8682-0481-2019

**Published:** 2020-10-05

**Authors:** Letícia Brito Mendes Pimenta, Nicole Zanzarini Sanson, Márcia Souza Volpe, Marcelo Britto Passos Amato, Adilha Misson Rua Micheletti, Luciana de Almeida Silva Teixeira

**Affiliations:** 1 Universidade Federal do Triângulo Mineiro, Programa de Pós-Graduação Stricto Sensu em Medicina Tropical e Infectologia, Uberaba, MG, Brasil.; 2 Universidade Federal do Triângulo Mineiro, Curso de Graduação em Medicina, Uberaba, MG, Brasil.; 3 Universidade Federal de São Paulo, Campus Baixada Santista, Departamento de Ciências do Movimento Humano, Santos, SP, Brasil.; 4 Universidade de São Paulo, Divisão Pulmonar, Departamento de Cardiopneumologia, São Paulo, SP, Brasil.; 5 Universidade Federal do Triângulo Mineiro, Departamento de Clínica Cirúrgica, Uberaba, MG, Brasil.; 6 Universidade Federal do Triângulo Mineiro, Departamento de Clínica Médica, Uberaba, MG, Brasil.

**Keywords:** Acute respiratory distress syndrome, Influenza virus, Mechanical ventilation

## Abstract

**INTRODUCTION:**

Patients with acute respiratory failure due to influenza require ventilatory support. However, mechanical ventilation itself can exacerbate lung damage and increase mortality.

**METHODS:**

The aim of this study was to describe a feasible and protective ventilation protocol, with limitation of the tidal volume to ≤6 mL/kg of the predicted weight and a driving pressure ≤15 cmH_2_O after application of the alveolar recruitment maneuver and PEEP titration.

**RESULTS:**

Initial improvement in oxygenation and respiratory mechanics were observed in the four cases submitted to the proposed protocol.

**CONCLUSIONS:**

Our results indicate that the mechanical ventilation strategy applied could be optimized.

Approximately 60-88% of patients admitted to intensive care units (ICUs) with respiratory complications resulting from influenza virus infection require mechanical ventilation. Among those patients who develop acute respiratory distress syndrome (ARDS), 21-48% die^1^. 

Mortality in patients with ARDS as a complication of influenza is mainly related to the difficulty in ventilating them. Severe hypoxemia refractory to conventional strategies of mechanical ventilation is frequently observed. Furthermore, divergences in ventilatory parameters are found, and there is no protocol that ensures protective mechanical ventilation to this population. There have been no clinical trials, and the results of case and protocol studies are inconclusive, especially regarding the use of alveolar recruitment maneuvers (ARM) and the adjustment of protective positive end-expiratory pressure (PEEP)^2^ ^,^ ^3^. 

In our institution, in the past three years, 20 patients with a confirmed diagnosis of influenza required mechanical ventilation. More than 80% of cases were ventilated with high tidal volumes (>8 mL/kg of the predicted body weight [PBW]) and fractions of inspired oxygen (FiO_2_) ≥0.6 for a period longer than 48 h. Recruitment maneuvers and titration of optimal PEEP were performed in only 35% of the cases, and the mean PEEP was 13 cmH_2_O. The mortality rate was 80%.

In light of this scenario, the objective of the present study was to describe the application of a feasible and protective mechanical ventilation strategy in patients with suspicion of ARDS caused by influenza. The mechanical ventilation strategy applied aimed to reduce mechanical stresses on the lung - minimization of alveolar collapse and hyperdistension - by using low tidal volumes, limiting the delta pressure, and setting the optimal PEEP after ARM. 

This study was approved by the Ethics Committee of Universidade Federal do Triângulo Mineiro (UFTM) (CAEE: 2.651.578) and was conducted at the 12-bed ICU of the university hospital of UFTM. The inclusion criteria were: age ≥18 years; suspicion of ARDS due to influenza (classification of the Brazilian Ministry of Health)^4^; use of oseltamivir for less than 48 h; and receiving mechanical ventilation for less than 48 h. The exclusion criteria were: failure of more than 3 organs evaluated using APACHE II; hemodynamic instability (mean arterial pressure <60 mmHg or need for noradrenaline >2 mg/kg/min); previous diagnosis of heart failure functional class 3 or 5; and acute brain injury.

Measurements of respiratory mechanics, ventilatory parameters, arterial blood gas parameters, length of ICU and hospital stay, duration of mechanical ventilation, and clinical outcomes at 28 days were evaluated. 

The protective strategy consisted of limiting the tidal volume to ≤6 mL/kg of the PBW and driving pressure (plateau pressure minus PEEP) to ≤15 cmH_2_O after the application of ARM and adjustment of PEEP according to the titrated value. During ARM and PEEP titration, all patients were sedated with fentanyl and midazolam, in addition to neuromuscular block with cisatracurium hydrochloride. ARM was performed in the pressure-controlled ventilation mode with a driving pressure of 15 cmH_2_O. An initial PEEP of 10 cmH_2_O was set, with increments of 5 cmH_2_O every minute until 30 cmH_2_O was reached, lasting 5 minutes. Decremental PEEP titration was then started at 25 cmH_2_O, with a decrease in PEEP of 2 cmH_2_O every minute until 5 cmH_2_O. Static compliance of the respiratory system was measured in each decremental step. At the end of PEEP titration, a new ARM was performed, and PEEP was adjusted to the value that produced the best respiratory compliance^5^, followed by adjusting the tidal volume at ≤6 mL/kg of the PBW and the driving pressure at ≤15 cmH_2_O. 

The patient was classified as responsive to the maneuver if a reduction in the driving pressure ≥3 cmH_2_O occurred. In the case of accidental disconnection of the ventilator or the patient required FiO_2_ ≥80% for oxygen saturation ≥92%, a new ARM and decremental PEEP titration were performed.

In the case of refractory hypoxemia (PaO_2_ ≤60 mmHg for at least 6-8 h in the presence of 100% FiO_2_), refractory acidosis (pH 7.1 for at least 1 h), or refractory barotrauma (persistent pneumothorax with 2 drains on the affected side or increase in subcutaneous or mediastinal emphysema with 2 chest drains), rescue therapy in the prone position for 16 h was used, accompanied by a new maximum recruitment maneuver and decremental PEEP titration, which was maintained until the PaO_2_/FiO_2_ ratio was ≥150 with FiO_2_ ≤0.6 and PEEP ≤10 cmH_2_O in the supine position.

For ventilatory weaning, we first reduced FiO_2_ to 0.4 and then PEEP by 2 cmH_2_O every 24 h until it was between 12-16 cmH_2_O. If the tidal volume of the patient was ≥ 9mL/kg of the PBW, weaning was not continued and administration of Precedex may have been necessary. Extubation occurred at a PEEP of 12 cmH_2_O and pressure support of 7 cmH_2_O. Noninvasive ventilation with a PEEP of 12 cmH_2_O was used in the first 24-48 h after extubation and was maintained as long as possible in the first 24 h, followed by progressive reduction. 

Between January 2018 and August 2019, 32 patients with suspected influenza were admitted to the UFTM university hospital, 12 of them required ventilatory support and 4 were eligible for the study.

The characteristics, mechanical ventilator parameters, and progression of the four patients are shown in Table 1. The APACHE II score for the evaluation of initial disease severity was 19 ± 6. All patients received oseltamivir within 2-5 days after the onset of symptoms. The two patients with a laboratory confirmation of influenza by RT-PCR died. During the ARM and PEEP titration, none of the patients exhibited hemodynamic alterations allowing completion of the protocol. Table 1 also shows the PaO_2_/FiO_2_ ratio and PEEP and driving pressure before and 2 h after the application of the protocol. An noticeable increase in the PaO_2_/FiO_2_ ratio was observed in three of the four patients, while patient 3 exhibited a slight reduction. The titrated PEEP was 13 and 17 cmH_2_O in the confirmed cases of influenza infection, while the values were much lower, 5 and 7 cmH_2_O, in the unconfirmed cases. Interestingly, except for patient 3, the titrated PEEP was lower than the initially adjusted value. With respect to lung recruitability, a driving pressure reduction by 2 cmH_2_O, immediately after the protocol application, was only possible in two cases. 

**TABLE 1:** Clinical characteristics and mechanical ventilator parameters of patients submitted to the protective mechanical ventilation protocol.

Parameters	Patient 1	Patient 2	Patient 3	Patient 4
Age (years)	63	52	65	68
Sex	M	F	M	M
Comorbidities	SAH, chronic AF, Hypothyroidism, RTV	SAH, obesity	Smoking: 15.9 pack-years	Hypogonadotropic hypogonadism, aortic and tricuspid regurgitation, hepatic steatosis, CRF, nephrolithiasis
APACHE II	25	17	9	25
Hemodialysis	Yes	Yes	No	No
Secondary infections	Bacterial PNM	Bacterial PNM	Bacterial PNM	Bacterial PNM
Vaccination	No	No	No	Yes
Influenza confirmation	No	Yes	No	Yes
Corticosteroids	Yes	Yes	No	Yes
Antiviral	Oseltamivir	Oseltamivir	Oseltamivir	Oseltamivir
Interval between symptom onset and beginning of antiviral use (days)	2	5	3	4
Ventilator parameters				
PEEP (cmH_2_O)				
Basal	14	16	12	15
2h after protocol completion	7	17	5	13
∆P (cmH_2_O)				
Basal	16	13	9	13
2 h after protocol completion	14	11	9	13
PaO_2_/FiO_2_				
Basal	83.8	58.0	192.0	91.4
2h after protocol completion	154	104.0	184.0	151.0
Number of ARM	3	3	1	3
Duration of MV (days)	25	17	6	4
Length of ICU stay (days)	26	17	12	5
Length of hospital stay (days)	61	17	30	5
Clinical outcome	Discharged	Death	Discharged	Death

Legend: **M:** male; **F:** female; **MV:** mechanical ventilation; **ARM:** alveolar recruitment maneuver; **ICU:** intensity care unit; **SAH:** systemic arterial hypertension; **AF:** atrial fibrillation;**RTV:** virus HIV;**CRF:** chronic renal failure;**PNM:** pneumonia; **PEEP:** positive end-expiratory pressure; **∆P:** driving pressure; **PaO**_2_ **/FiO**_2:_ ratio of partial pressure arterial oxygen and fraction of inspired oxygen.

Regarding static and dynamic compliance, patients who died had substantially lower values than the normal range. The plateau pressure remained within the protective limits, except for the last measurement of patient 4 moments before death. Airway resistance remained high in two cases (Table 2). Despite improvements of the PaO_2_/FiO_2_ ratio, the respiratory mechanics of these patients were compromised, a fact that may be associated with the physiopathogenesis of the disease and its progression to death.

**TABLE 2:** Parameters of respiratory mechanics in patients who died.

Parameter	Before protocol		After 24 h		After 48 h		After 72 h	
	Patient 2	Patient 4	Patient 2	Patient 4	Patient 2	Patient 4	Patient 2	Patient 4
Cst (mL/cmH_2_O)	29	25	26	20	30	26	38	21
RR: 60-100								
Cdyn (mL/cmH_2_O)	23	21	23	24	25	18	31	16
RR: 50-80								
Raw (cmH_2_O/l/s)	14	19	13	23	17	15	16	16
RR: 2-5								

Legend: **Cst**: static compliance; **Cdyn**: dynamic compliance; **AWR:** airway resistance; **RR:** reference range. Source: Opasich et al^6^.

Oxygenation continued to improve in patients 1, 2, and 3, but not in patient 4. Patient 2 showed marked improvement in the first 5 days, but important clinical worsening was observed after accidental extubation, and on day 17 she died. The driving pressures were maintained within the protective range in all cases. 

Figure 1 shows the lung images of patients 2 and 4 before and after the mechanical ventilation protocol was applied. There was a marked improvement on the chest X-ray of patient 2, but not patient 4. The use of a real-time lung monitoring tool such as electrical impedance tomography may have helped to individualize the mechanical ventilation strategy applied to patient 4, allowing for better resolution of lung collapse/consolidation (Figure 1). 

**FIGURE 1:**
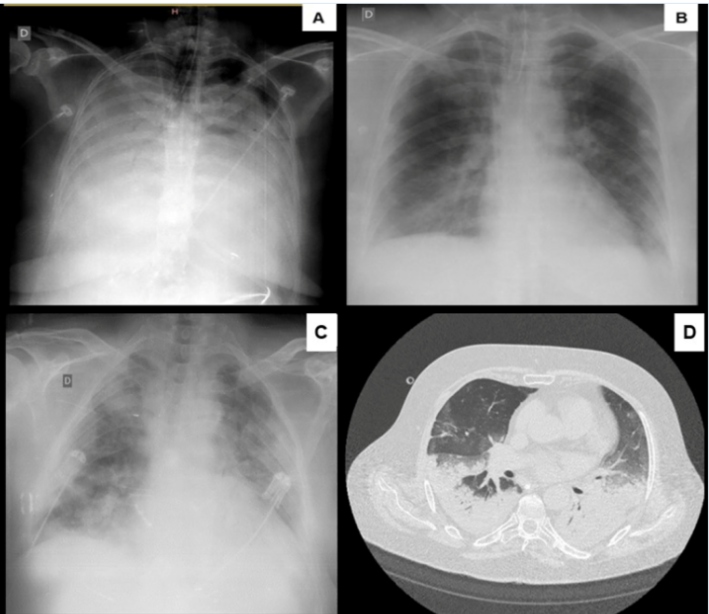
Chest images of patient 2 (**A and B**) and patient 4 (**C and D**). **A** and **C** images were obtained before the protective mechanical ventilation protocol was started and **B** and **D** images 24 to 48 h after initiation of the protocol. Note that patient 2 showed a marked improvement on chest X-ray after the protocol was applied, whereas patient 4 did note. Computed tomography scan (**D**) showed extensive lower lobe consolidations related to the inflammatory/infectious process associated with ground-glass attenuation, which may represent an edema component.

Influenza A virus is an important causative agent of acute respiratory disease^3^, which can result in ARDS and the need for ventilatory support. However, studies confirm that mechanical ventilation can exacerbate preexisting lung damage or even cause pulmonary injury itself^7^. This injury occurs during alveolar hyperdistension and cyclic opening and collapse of the alveoli. In addition, the mechanical stimulus is converted to biomolecular activity through mechanotransduction, which triggers the release of a range of inflammatory mediators and neutrophil infiltration. Ultimately, these events lead to multi-organ dysfunction and death^8^. 

Cornejo et al.^2^ and Venkategowda et al.^3^ demonstrated that protective mechanical ventilation strategies using low tidal volumes improved outcomes among patients with ARDS after influenza infection. In addition, high levels of PEEP and ARM have been used to improve oxygenation, opening collapsed alveoli, and allowing a more homogenous distribution of ventilation^2^ ^,^ ^9^. Analysis of recruitability in the present study showed that it was not possible in any of the cases to reduce the driving pressure by 3 cmH_2_O, the reference value defined for responsiveness to recruitment. A possible explanation for this finding is that the patients already exhibited safe driving pressures before the start of the protocol. Evidence indicates that patients with high driving pressures respond best to protective strategies^10^. However, initial improvement in oxygenation was observed in most patients. The optimal PEEP found in the confirmed cases was relatively high (13 and 17 cmH_2_O), similar to other reports^4^ ^,^ ^8^. It must be stressed that in three patients the titrated PEEP was lower than the initially adjusted PEEP, a finding which illustrated the complexity of protective ventilation in these patients. 

Both patients with confirmed infection died. The presence of comorbidities is recognized as a risk factor for severe outcomes in patients infected with influenza^11^. Comorbidities were found in all patients of the study. The early initiation of treatment with antiviral medication (within 48 h) is considered a protective factor^12^. In the patients with fatal outcomes, treatment was initiated late (about 4-5 days after the onset of symptoms).

Laboratory tests for the diagnosis of influenza have limitations that can produce misleading results. In our cases, RT-PCR was used, which shows a sensitivity around 80.0% and specificity of 95%^13^. However, we cannot definitively rule out that patients without laboratory confirmation did not actually have influenza viral infection. We therefore included two patients without laboratory confirmation.

Despite the small sample size, the reported findings are considered relevant. There is a lack of trials on mechanical ventilation involving patients with influenza and this study provides some insight on how to apply a protective mechanical ventilation strategy to this population. The use of a real-time lung monitoring tool to individualize the ventilation strategy, such as electrical impedance tomography, may help to optimize the proposed strategies.
